# Gradient Chitosan Hydrogels Modified with Graphene Derivatives and Hydroxyapatite: Physiochemical Properties and Initial Cytocompatibility Evaluation

**DOI:** 10.3390/ijms21144888

**Published:** 2020-07-10

**Authors:** Karolina Kosowska, Patrycja Domalik-Pyzik, Małgorzata Sekuła-Stryjewska, Sylwia Noga, Joanna Jagiełło, Magdalena Baran, Ludwika Lipińska, Ewa Zuba-Surma, Jan Chłopek

**Affiliations:** 1 Department of Biomaterials and Composites, Faculty of Materials Science and Ceramics, AGH University of Science and Technology, 30-059 Krakow, Poland; kosowska@agh.edu.pl (K.K.); chlopek@agh.edu.pl (J.C.); 2 Malopolska Centre of Biotechnology, Jagiellonian University, 30-387 Krakow, Poland; malgorzata.sekula@uj.edu.pl (M.S.-S.); sylwia.noga@doctoral.uj.edu.pl (S.N.); 3 Department of Cell Biology, Faculty of Biochemistry, Biophysics and Biotechnology, Jagiellonian University, 30-387 Krakow, Poland; ewa.zuba-surma@uj.edu.pl; 4 Department of Chemical Synthesis and Flake Graphene, Łukasiewicz Research Network—Institute of Electronic Materials Technology, 01-919 Warsaw, Poland; joanna.jagiello@itme.edu.pl (J.J.); magdalena.baran@itme.edu.pl (M.B.); Ludwika.Lipinska@itme.edu.pl (L.L.)

**Keywords:** chitosan, graphene family materials, hydrogels, gradient nanocomposites

## Abstract

In this study, we investigated preparation of gradient chitosan-matrix hydrogels through a novel freezing–gelling–thawing method. The influence of three types of graphene family materials (GFM), i.e., graphene oxide (GO), reduced graphene oxide (rGO), and poly(ethylene glycol) grafted graphene oxide (GO-PEG), as well as hydroxyapatite (HAp) on the physicochemical and biological properties of the composite hydrogels was examined in view of their potential applicability as tissue engineering scaffolds. The substrates and the hydrogel samples were thoroughly characterized by X-ray photoelectron spectroscopy, X-ray diffractometry, infrared spectroscopy, digital and scanning electron microscopy, rheological and mechanical analysis, in vitro chemical stability and bioactivity assays, as well as initial cytocompatibility evaluation with human umbilical cord Wharton’s jelly mesenchymal stem cells (hUC-MSCs). We followed the green-chemistry approach and avoided toxic cross-linking agents, using instead specific interactions of our polymer matrix with tannic acid, non-toxic physical cross-linker, and graphene derivatives. It was shown that the most promising are the gradient hydrogels modified with GO-PEG and HAp.

## 1. Introduction

Chitosan (CS) is a well-known linear, semi-crystalline polysaccharide, the second most widespread biopolymer. The physicochemical and biological properties of a particular chitosan are governed by a degree of deacetylation (DD), i.e., the ratio of chitosan main building blocks d-glucosamine and *N*-acetyl-d-glucosamine [1,2,3,4]. The DD shows information on the amount of monomeric units equipped with amino groups and these groups are responsible for many unique characteristics of chitosan, such as its cationic nature or ability to dissolve in water under mild acidic conditions [5,6]. Amino and hydroxyl groups of chitosan chain can be exploited to tailor specific polymer properties or introduce additional functionalities. Chitosan resemblance to mammalian polysaccharides and ability to form hydrogels combined with other characteristics, make it a very promising biomaterial in many applications [7,8,9,10]. Some recent studies include preparation of composite scaffolds based on chitosan functionalized with boronic acid [11], bone biomicroconcretes containing hybrid hydroxyapatite-chitosan granules [12], chitosan encapsulation of bioactive molecules [13], amphiphilic micelles for cancer therapy based on CS grafted with *O*-methyl-*O*′-succinylpolyethylene glycol and oleic acid [14], or even a potential application of *N*-(2-hydroxypropyl)-3-trimethylammonium chitosan chloride (HTCC) as inhibitor of some current coronaviruses [15].

In biomaterials science, it is usually best to follow natures example, i.e., apply a biomimetic approach. With this in mind, tissue engineering scaffolds should have gradient structure, similarly to natural tissues. The term ‘gradient’ can be referred to a material composition, mechanical properties (e.g., gradient of Young’s modulus) or bioactive signals presence. Hydrogels, defined as three-dimensional (3D) polymer networks with high water content, are often primary materials for further modifications. However, typically they are fabricated from homogenous solutions what results in formation of isotropic gels. Gradient hydrogels can be obtained using more sophisticated methods such as 3D printing or microfluidics, but these are time-consuming and require specialized equipment. Alternatively, gradient systems may be produced through carefully optimized gelation [16]. 

Chitosan hydrogels being soft and pliable, often lack appropriate mechanical properties for some more demanding applications, e.g., bone tissue engineering. Their mechanical strength can be largely improved through formation of hybrid composites modified with graphene-based materials [17,18,19,20]. The last few years have been truly ‘the rise of graphene’ as Geim and Novoselov stated [21]. Undoubtedly, it is due to the fact that graphene family materials (GFM) [22] are unique in so many ways. From graphene, a one-atom-thick sheet of sp^2^-bonded carbon atoms arranged in a honeycomb lattice, to its various derivatives produced by chemical or physical modifications, GFM are known for their exceptional mechanical properties, high thermal and electrical conductivity, high chemical stability, high optical transmittance, large surface area, and many other, often tunable, properties that are exploited in vast number of applications [23,24,25]. However, in biomedical fields, two of them, i.e., graphene oxide (GO) and reduced graphene oxide (rGO), are of particular interest. 

In this study, a freezing–gelling–thawing method was developed for fabrication of bulk gradient chitosan-matrix hydrogels. Moreover, the influence of three types of graphene family materials, i.e., GO, rGO, and poly(ethylene glycol) grafted graphene oxide (GO-PEG), as well as hydroxyapatite (HAp) on the physicochemical and biological properties of the composite hydrogels was examined in view of their potential applicability as tissue engineering scaffolds. We followed green-chemistry approach and avoided toxic cross-linking agents, using instead specific interactions of our polymer matrix with tannic acid, non-toxic physical cross-linker, and graphene derivatives. As properties of polymer nanocomposites rely heavily on the dispersion of nanofillers in a matrix, we focused our attention also on fabrication issues.

## 2. Results and Discussion

### 2.1. Characterization of the Graphene Materials

**Figure 1:**
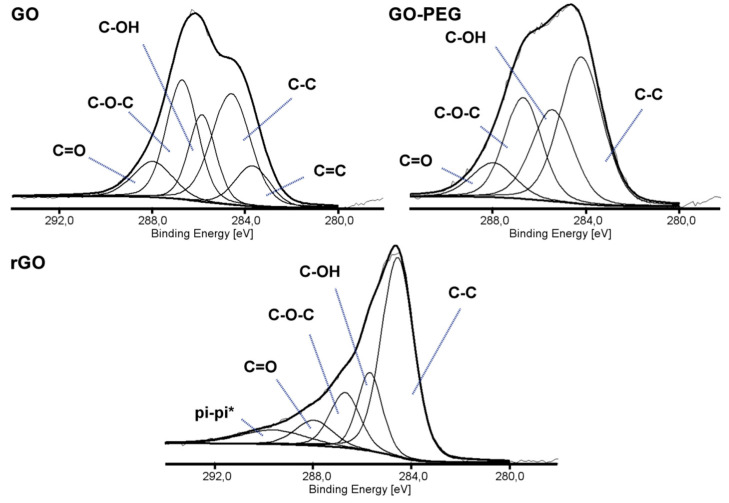
X-ray photoelectron spectroscopy (XPS) spectra (C1s) of graphene oxide (GO), poly(ethylene glycol) grafted graphene oxide (GO-PEG) and reduced graphene oxide (rGO).

Three types of graphene-based nanofillers were analyzed by X-ray photoelectron spectroscopy (XPS) to study the chemical states and the presence of the functional groups. Figure 1 shows the high energy resolution spectra of the C1s region. The deconvoluted spectrum of GO showed five peaks of the following binding energies: 283.68, 284.60, 285.85, 286.71, and 287.98 eV, corresponding to five types of carbon components: C=C, C-C/C-H, carbon of C-OH, carbon of C-O-C and the carbon of C=O, respectively [26,27]. Decrease of the intensity of the peaks assigned to functional groups containing oxygen and the presence of the peak at 289.67 eV assigned to π-π* satellite bonds in rGO spectra, suggested partial reconstruction of a graphene structure. Additionally, changes in the C1s/O1s atomic ratio, calculated from the XPS spectra, confirmed the reduction in the number of oxygen-containing functional groups. For GO, the ratio was 0.95, while for rGO 1.79. The grafting of graphene oxide surface with poly(ethylene glycol) was carried out to improve the dispersion of GO in a polymer matrix and the adhesion between the nanofiller and the chitosan. The C1s/O1s atomic ratio remained the same as for GO (0.95), but the peak intensity from C-O-C binding, which builds the PEG structure, increased.

**Figure 2:**
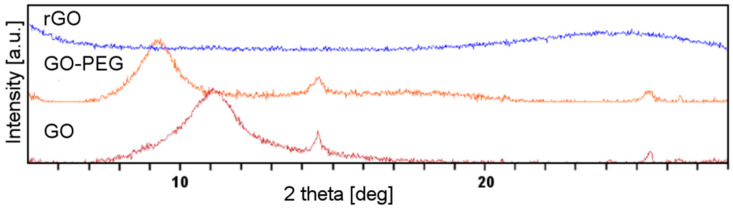
X-ray diffractometry (XRD) patterns of graphene oxide (GO), poly(ethylene glycol) grafted graphene oxide (GO-PEG) and reduced graphene oxide (rGO).

Another tool commonly used to investigate the graphene structure is X-ray diffractometry (XRD). The Bragg’s law allows the calculation of the d-spacing between the graphene sheets. The oxygen-containing functional groups increase the d-spacing in graphene. Additionally, their hydrophilic character facilitates absorption of water molecules, and thus further increases the d-spacing. Figure 2 shows the XRD pattern of GO, rGO and GO-PEG. The sharp diffraction peaks observed at 11.06° (d-spacing: 0.80 nm) correspond to the plane of hexagonal crystalline structure of GO. After reduction, a new, wide peak showed up at 24.54°, corresponding to the inter layer distance of 0.36 nm, a value very similar to d-spacing in graphene. In the case of GO-PEG, the peak shifted towards a smaller deflection angle as compared to GO (9.26° vs. 11.06°). Grafting of the GO surface with PEG resulted in an even greater increase of the distance between graphene sheets than in GO (0.95 nm). Hence, interactions between layers were weakened.

### 2.2. Rheological Characterization of Hydrogels

One of the reasons behind introducing graphene family materials into polymer matrices was to create composite materials with favorable mechanical properties. However, the improvement in the mechanical properties depends largely on the homogenous distribution of the nanofillers in the polymer matrix [28]. Interfacial interactions play a key role here. If the particles are poorly dispersed, agglomerates are formed and defects occur in the matrix. Graphene tends to agglomerate due to a large surface area and strong van der Waals interactions, which in turn negatively affects the transfer of stress between the phases of the composite.

**Figure 3:**
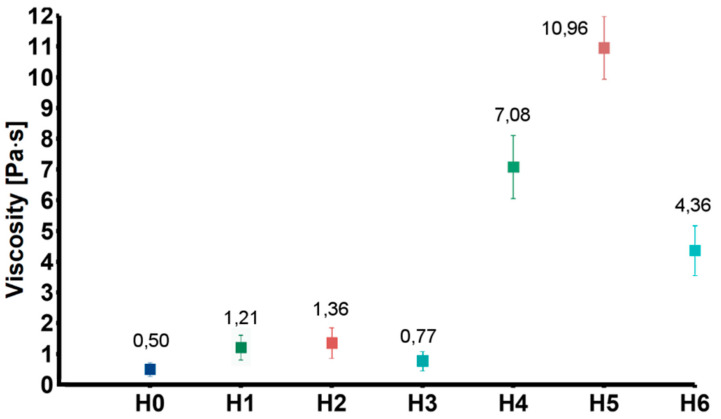
Viscosity of the tested solution (H0: chitosan (CS)) and dispersions (H1: CS/graphene oxide (GO); H2: CS/poly(ethylene glycol) grafted graphene oxide (GO-PEG); H3: CS/reduced graphene oxide (rGO); H4: CS/GO/hydroxyapatite (HAp); H5: CS/GO-PEG/HAp; H6: CS/rGO/HAp).

To obtain information on the interactions between the nanofillers and the chitosan chains, rheological measurements were carried out. Figure 3 shows values of viscosity of the tested samples obtained at 20 Hz (representative for the entire measured frequency range). The viscosity of the solutions modified with GO (H1) and GO-PEG (H2) were higher as compared to the CS solution (H0). This was probably due to weak interactions between oxygen-containing functional groups of GO and chitosan amino groups. Additionally, chitosan and PEG can form strong inter-chain interactions by ionic complexation between chitosan amino groups and PEG hydroxyl groups. As expected, addition of rGO (H3), in which the number of functional groups was reduced, caused only slight increase of viscosity. Much higher viscosity values were observed in case of the group modified also with HAp (H4–H6). The increase in viscosity resulted from relatively high content of ceramic particles. 

### 2.3. Microstructure

**Figure 4:**
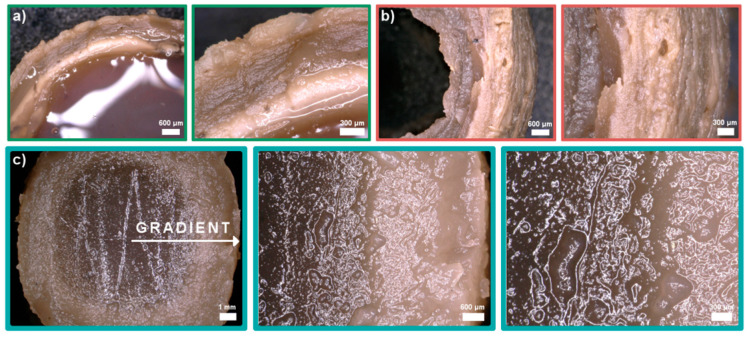
Digital microphotographs of chitosan/graphene oxide (H1) hydrogels obtained in three types of cross-linking solutions: (**a**) 5% TPP (sodium tripolyphosphate), 2 h + 10% NaOH, 10 min, (**b**) 5% TPP, 4 h + 10% NaOH, 10 min, (**c**) 0.5% TPP with 5% NaCl, 24h.

Cross-linking of the samples was optimized with different solutions and gelling times: (i) 5% TPP (sodium tripolyphosphate), 2 h + 10% NaOH, 10 min; (ii) 5% TPP, 4 h + 10% NaOH, 10 min, (iii) 0.5% TPP with 5% NaCl, 24 h. Morphology of the obtained samples varied significantly (Figure 4). When 5% TPP and 10% NaOH solutions were used, a multilayer hydrogel structure was observed (Figure 4a,b). Nie and others explained this phenomenon by the process of creating Liesegang rings [29]. Unfortunately, the samples melted before forming a strong outer layer causing the hydrogel to lose its shape. Additionally, without using a second bath in 10% NaOH, the samples deformed completely after removal from the 5% TPP solution. The use of NaCl increased ionic strength of the cross-linking solution. As a result, the diffusion of Cl^-^ ions into the interior of the hydrogel allowed the cross-linking time to be extended. Gradually the Cl^-^ ions were replaced by TPP and the hydrogel with a gradient structure was formed (Figure 4c). 

**Figure 5:**
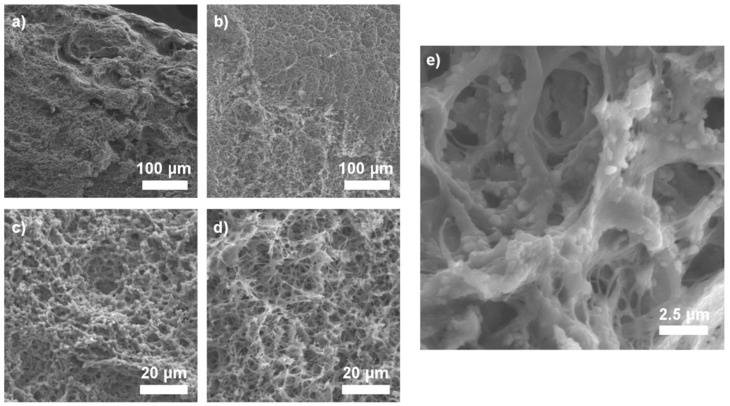
Representative SEM images of gradient hydrogels after freeze-drying (sample H5: chitosan/poly(ethylene glycol) grafted graphene oxide (GO-PEG)): (**a**) Edge of the sample, (**b**) Gradient inside, (**c**) Outer part, (**d**) Inner part, (**e**) Surface with visible hydroxyapatite (HAp) particles.

Figure 5 shows microstructure of the freeze-dried H5 hydrogel (i.e., the sample modified with 0.5% GO-PEG and 10% HAp). The surface of the hydrogel was gradient and made of three layers. The outer layer (edge) was jagged and very porous. Ficture 5b shows the transition zone. The pores formed a network throughout the composite. Closer to the edge of the sample (where cross-linking density was higher) the pores were smaller and the surface was less rough. Inside the sample, the pore walls were narrow and the pores formed a dense network. 

The difference in the size and shape of the pores outside and inside is the result of a difference in cross-linking speed. Inside the sample, the cross-linking speed of the frozen solution in the cross-linking bath was lower than speed of the melting. Hence, more solution remained uncross-linked, which after freeze drying resulted in larger pores (20–50 µm) and thinner walls.

Figure 5e shows well dispersed hydroxyapatite particles, which is important both for mechanical properties and for matrix crosslinking. We can speculate that large agglomerates of the ceramic particles would hinder effective crosslinking. 

### 2.4. Cross-Linking Degree

**Figure 6:**
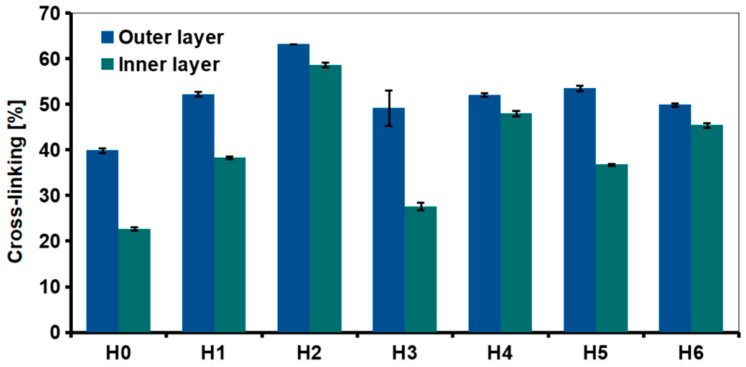
Degree of cross-linking in chitosan-based hydrogels (H0: chitosan (CS); H1: CS/graphene oxide (GO); H2: CS/poly(ethylene glycol) grafted graphene oxide (GO-PEG); H3: CS/reduced graphene oxide (rGO); H4: CS/GO/hydroxyapatite (HAp); H5: CS/GO-PEG/HAp; H6: CS/rGO/HAp) measured by ninhydrin assay.

The degree of cross-linking of the outer and inner layers was examined using ninhydrin assay (Figure 6). The test was also carried out to evaluate the effect of the nanofillers and HAp addition. Interactions between the chitosan amino groups with graphene-based materials and hydroxyapatite should result in a higher degree of cross-linking as compared to H0. A significant difference in the degree of chitosan cross-linking between the center of the sample and the outer layer was measured in each hydrogel. For the reference sample, the degree of cross-linking of the outer layer was about 40% and the inner layer about 22%. 

The introduction of 0.5% of the nanofillers significantly decreased the amount of free amino groups in chitosan, thus higher cross-linking degree was observed, e.g., slightly above 60% in case of the outer layer of GO-PEG—modified, H2 sample. This suggested that grafting GO surface with PEG improved interfacial adhesion between chitosan and GO [28]. Of the three types of graphene derivatives, reduced graphene oxide was the least effective. In rGO, part of oxygen-containing functional groups that may interact with chitosan functional groups was removed. It also resulted in stronger interactions between the graphene sheets, because d-spacing was reduced. The adhesion of polymer chains was weaker due to reduction of the surface area. Removal of some functional groups also resulted in a greater tendency to agglomeration.

Addition of HAp also affected cross-linking degree but its effect was dependent on the graphene derivative used in the composite system. For the outer layer of all the HAp-containing samples (H4–H6), cross-linking was similar—around 50%, it was also higher than for the reference, H0 sample. The amount of free amino groups for HAp samples was similar or lower than for respective hydrogels modified only with GO or rGO (H4 and H6 vs H1 and H3). Interestingly, when comparing H2 and H5 (GO-PEG vs GO-PEG/HAp modification, respectively), it is clear that the introduction of ceramic particles into the polymer matrix resulted in lower cross-linking efficiency. 

### 2.5. Chemical Stability

**Figure 7:**
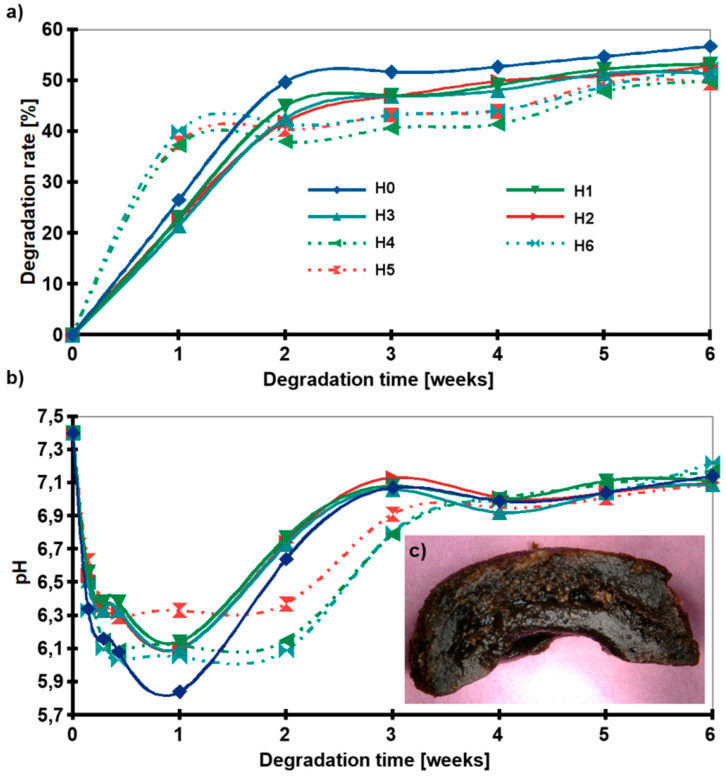
Degradation behavior: (**a**) Weight loss of the samples as a function of incubation time in PBS, (**b**) pH of media, (**c**) Digital microphotograph of H2 after 6 weeks of incubation (slice cut in half); (H0: chitosan (CS); H1: CS/graphene oxide (GO); H2: CS/poly(ethylene glycol) grafted graphene oxide (GO-PEG); H3: CS/reduced graphene oxide (rGO); H4: CS/GO/hydroxyapatite (HAp); H5: CS/GO-PEG/HAp; H6: CS/rGO/HAp).

A cross-linking gradient was obtained to control the degradation of the hydrogel. The positive effect of the nanofillers addition on the stability of the hydrogels in PBS media was observed during the incubation test. Figure 7a shows the weight loss of the samples. In the first step, the degradation was rapid. During the first two weeks of incubation, most of the lactic acid was removed from the samples and the pH dropped from 7.4 to about 6 (Figure 7b). In the next step, the pH of the medium returned to neutral value and the pattern plateaued. Interestingly, for H1–H3 samples the weight loss after the first week of degradation was ≈20%, while for H4–H6 (hydrogels with HAp) ≈35%. However, after six weeks of degradation, the weight loss of all composite samples (H1–H6) was ≈50% and 57% for H0. All samples began to degrade from the inside and empty space was created (Figure 7c). 

### 2.6. Structural Analysis

**Figure 8:**
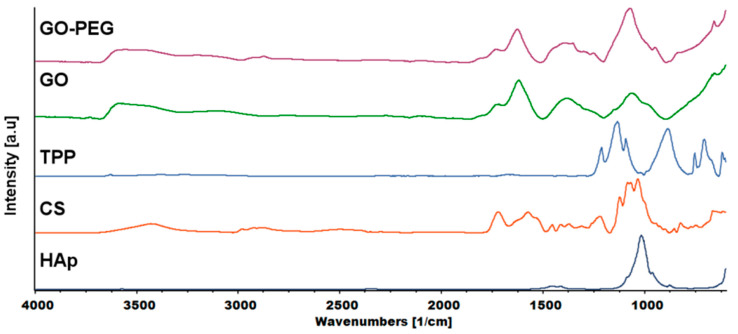
FTIR (Fourier transform infrared spectroscopy) spectra of the hydrogels components: HAp—hydroxyapatite, CS—chitosan, TPP—sodium tripolyphosphate, GO—graphene oxide, GO-PEG poly(ethylene glycol) grafted graphene oxide).

FTIR analysis was carried out to investigate the mechanism of interaction between chitosan and cross-linkers—graphene-derivatives, TPP and tannic acid (TAc). Figure 8 shows the spectra of chitosan, GO, GO-PEG and the cross-linker, TPP. Tannic acid (TAc) was also introduced into the hydrogels. Our previous research confirmed that TAc addition increases the mechanical properties of chitosan [19]. TAc has a very complex structure with many phenyl groups. They can form hydrogen bonds with -OH and –NH_2_ groups from chitosan. In addition, non-covalent van der Waals interactions may also be formed [30]. The characteristic peaks of hydrogel components are shown in Table 1. 

The characteristic peaks of CS appeared at ≈3400, ≈2800 and 1576 cm^−1^, which corresponded to the N-H stretching vibration overlapped with O-H stretching vibration, C-H asymmetric and symmetric stretching vibration and N-H bending vibration in primary amine, respectively. The peak at 1719 cm^−1^ was attributed to C=O stretching vibration, while the peak at 1120 cm^−1^ to asymmetric stretching vibration of C-O-C bridge [31]. The characteristic peaks of TPP at 1212, 1136 and 884 cm^−1^ corresponded to P=O stretching vibration, asymmetric and symmetric vibration in O-P=O groups and asymmetric stretching vibration of P-O-P bridge, respectively [32]. 

**Figure 9:**
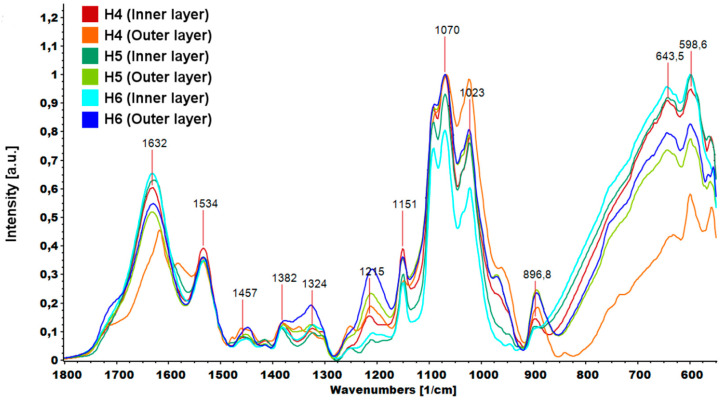
Parts of FTIR (Fourier transform infrared spectroscopy) spectra of hydrogels (H4: chitosan (CS)/graphene oxide (GO)/hydroxyapatite (HAp); H5: CS/ poly(ethylene glycol) grafted graphene oxide /HAp; H6: CS/reduced graphene oxide/HAp) in the range 1800–550 cm^−1^.

The graphene oxide spectrum provided information about oxygen-containing functional groups attached to the nanomaterial surface. The characteristic stretching vibration bands of O-H, carboxyl C=O and C-O were observed at ≈3400, 1725 and 1073 cm^−1^. The peak at 1626 cm^−1^ could be assigned to C=C skeletal vibration and the peak at 1391 cm^−1^ to C-OH deformation [33]. In the GO-PEG spectra, peaks originating from GO were visible, but slight shifts and changes in relative intensity were observed. In particular, the intensity of the peak attributed to C-O vibration increased significantly, which confirmed that PEG was grafted onto the surface of GO. The FTIR spectra of inner and outer layers of hydrogels (H4–H6) are shown in Figure 9. The peak of N-H bending vibration shifted from 1576 to 1632 cm^−1^ and a new peak assigned to N-O-P stretching vibration appeared at 1534 cm^−1^. This indicates that -NH groups of chitosan were cross-linked by ions from TPP. 

**Figure 10:**
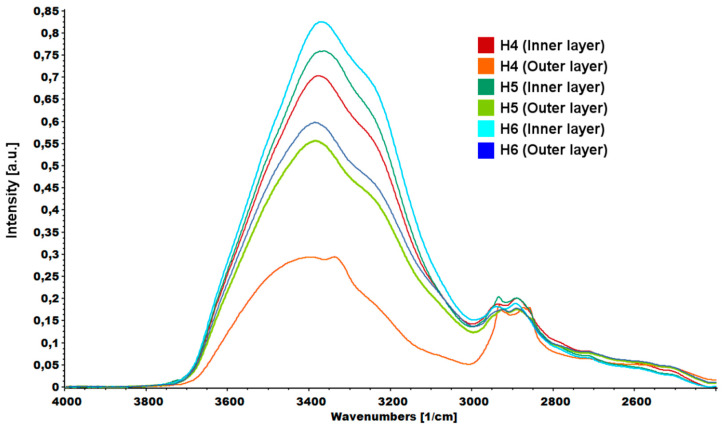
FTIR spectrum of hydrogels (H4: chitosan (CS)/graphene oxide (GO)/hydroxyapatite (HAp); H5: CS/ poly(ethylene glycol) grafted graphene oxide /HAp; H6: CS/reduced graphene oxide/HAp) peaks assigned to –OH vibrations.

An interesting relationship was noticed in the area assigned to vibrations from hydroxyl groups (Figure 10). Spectra were normalized according to the highest peak. In each case, the –OH peak measured for the inner layer of the hydrogel had a higher intensity than the peak measured for the outer layer. Higher peak intensity was due to the higher water content inside the hydrogels and weaker cross-linking.

### 2.7. Mechanical Properties

**Figure 11:**
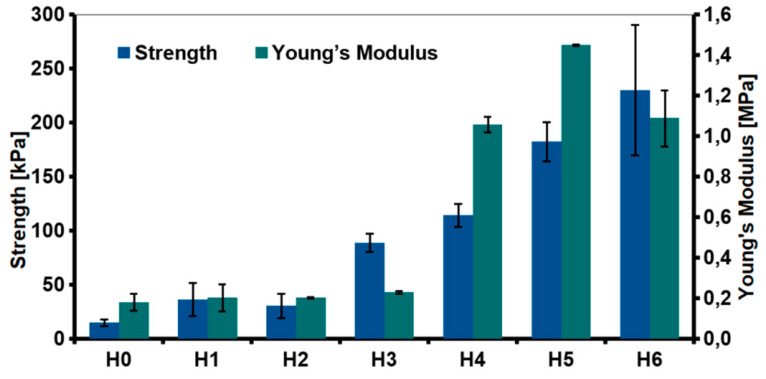
Mechanical properties (compressive strength and Young’s modulus) of the hydrogels (H0: chitosan (CS); H1: CS/graphene oxide (GO); H2: CS/poly(ethylene glycol) grafted graphene oxide (GO-PEG); H3: CS/reduced graphene oxide (rGO); H4: CS/GO/hydroxyapatite (HAp); H5: CS/GO-PEG/HAp; H6: CS/rGO/HAp).

The relationship between the type of the fillers and mechanical properties of the hydrogels was investigated through the compression test (Figure 11). The addition of the nanofillers improved the compression strength. The highest increase was observed for the hydrogel modified with rGO (88.79 ± 8.33 kPa for H3 as compared to unmodified H0 sample 15.02 ± 3.21 kPa). Various phenomena resulting from the introduction of the nanofillers into the chitosan matrix affected the mechanical properties. CS/rGO hydrogel had lower viscosity compared to CS/GO. Our previous research showed that rGO sheets tend to arrange parallel to each other due to the π-π* interactions [19]. As result of the interfacial adhesion, chitosan chains may have been blocked between the graphene sheets, what led to a significant increase in the compressive strength.

Even greater improvement in mechanical properties was observed for hydroxyapatite-modified hydrogels. The obtained results were correlated with measurements of the viscosity of the solutions used for the hydrogels preparation. Generally speaking, the proper stress transfer from the matrix to the reinforcing phase regulates mechanical properties of composites. The enhanced strength observed for H4–H6 hydrogels was probably due to the complexation of the HAp with chitosan and a good dispersion of those ceramic particles in the polymer matrix. The presence of organic acid may cause dissociation of hydroxyapatite and the formation of chelated calcium ions [34]. Hence, the chemical interaction caused by the formation of a coordination bond between Ca^2+^ ions and amine ions from chitosan. The FTIR spectra showed a shift in the peak attributed to N-H vibration, but this was due to both, graphene and hydroxyapatite interactions with chitosan. The most significant increase in Young’s module was measured for the hydrogel modified with GO-PEG (H5). It is expected that chitosan and PEG form strong inter-chain interactions by ionic complexation between chitosan amine and hydroxyl groups of PEG. 

### 2.8. In Vitro Bioactivity

**Figure 12:**
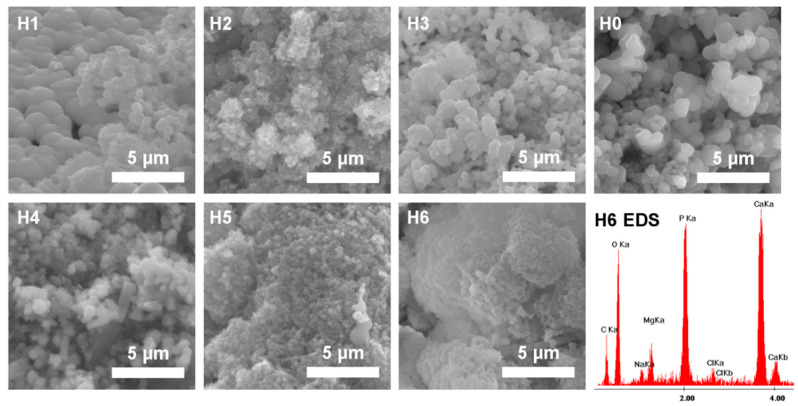
SEM (scanning electron microscopy) images of the hydrogels surface (H0: chitosan (CS); H1: CS/graphene oxide (GO); H2: CS/poly(ethylene glycol) grafted graphene oxide (GO-PEG); H3: CS/reduced graphene oxide (rGO); H4: CS/GO/hydroxyapatite (HAp); H5: CS/GO-PEG/HAp; H6: CS/rGO/HAp) after 4 weeks of incubation in simulated body fluid (SBF) solution and representative energy dispersive spectroscopy (EDS) spectrum (for H6).

The ability of the nanocomposites to promote a formation of an apatite-like layer on the surface was tested in the in vitro bioactivity test (incubation in simulated body fluid (SBF) solution). The assay is often used to predict bone-binding ability of the developed materials. Figure 12 shows SEM images of the hydrogel surfaces after four weeks of incubation. Spherical crystals were formed on the surface of all samples, but their morphology varied. The surface area covered with the apatite layer and the Ca/P ratio (based on EDS analysis) increased with incubation time. After 4 weeks, the Ca/P ratio was higher and more similar to the typical bone HAp (i.e., Ca/P around 1.67). The Ca/P ratio for H0–H3 was in the range of 1.26–1.35, while for H4–H6 it was 1.39–1.87. 

### 2.9. Cytocompatibility

#### 2.9.1. The Influence of Liquid Extracts of Chitosan on hUC-MSCs Morphology

**Figure 13:**
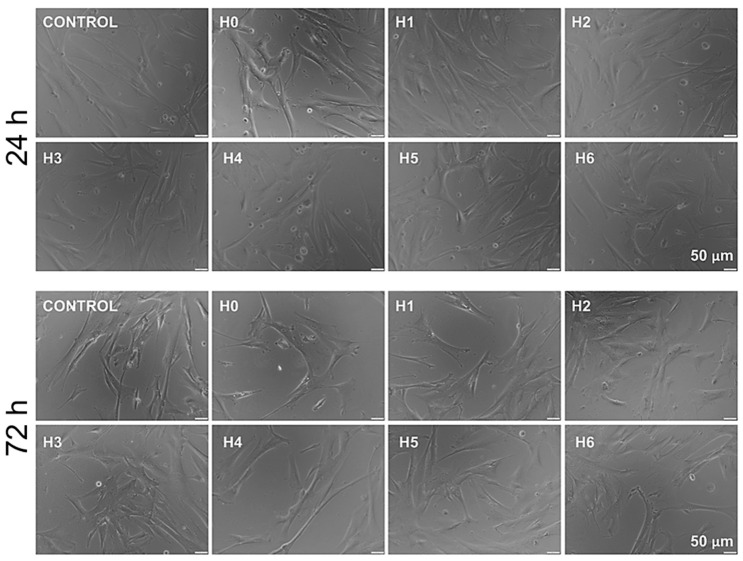
Representative images of morphology of hUC-MSCs (human umbilical cord Wharton’s jelly mesenchymal stem cells) cultured for 24 and 72 h in a particular liquid extract of chitosan-based samples (H0: chitosan (CS); H1: CS/graphene oxide (GO); H2: CS/poly(ethylene glycol) grafted graphene oxide (GO-PEG); H3: CS/reduced graphene oxide (rGO); H4: CS/GO/hydroxyapatite (HAp); H5: CS/GO-PEG/HAp; H6: CS/rGO/HAp). Scale bar: 50µm.

The morphology of hUC-MSCs (human umbilical cord Wharton’s jelly mesenchymal stem cells) cultured in liquid extracts of chitosan was evaluated after 24 h and 72 h of the cell culture. We observed no effect of analyzed extracts on cell morphology. hUC-MSCs exhibited typical, elongated fibroblast-like structure. Representative images are presented in Figure 13. 

#### 2.9.2. The Impact of Liquid Extracts of Chitosan on hUC-MSCs Cytotoxicity

**Figure 14:**
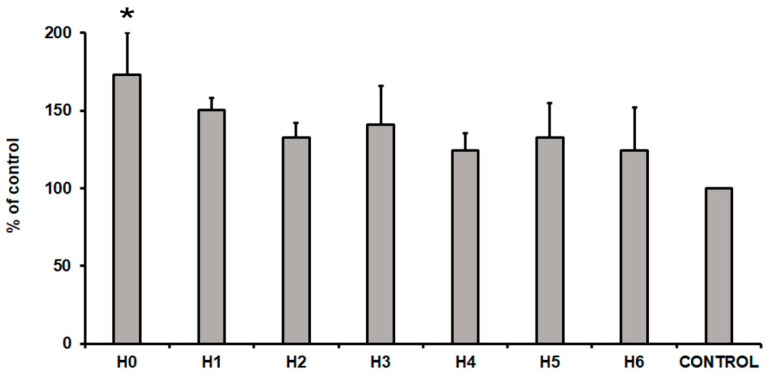
The cytotoxicity of liquid extracts of chitosan-based samples (H0: chitosan (CS); H1: CS/graphene oxide (GO); H2: CS/poly(ethylene glycol) grafted graphene oxide (GO-PEG); H3: CS/reduced graphene oxide (rGO); H4: CS/GO/hydroxyapatite (HAp); H5: CS/GO-PEG/HAp; H6: CS/rGO/HAp) towards hUC-MSCs (human umbilical cord Wharton’s jelly mesenchymal stem cells) after 24 h of the cell culture. Data are expressed as mean ± SD. P value less than 0.05 (*p* < 0.05) was considered as statistically significant and labeled by an asterisk (*).

After 24 h of hUC-MSCs culture in a particular liquid extract of chitosan the evaluation of their cytotoxicity was performed. To measure the cytotoxicity effect, a commercially available cytotoxicity detection kit was used. This method is based on the detection of the lactate dehydrogenase (LDH) in the cell culture medium. The LDH is a soluble cytoplasmic enzyme that is released into extracellular space when the cell plasma membrane is damaged. Thus, the level of the LDH released from cells is related with the cytotoxicity effect. The obtained results presented an increase level of the LDH in all the tested conditions (Figure 14). The highest level of the LDH (73% more than cells cultured in control medium) was observed in cells cultured in H0 extract (CS-only). This result indicated that extract from pure chitosan exhibited the highest cytotoxicity towards hUC-MSCs cells. Interestingly, CS modification with GO/GO-PEG/rGO (H1, H2, and H3 respectively) reduced cytotoxicity by about 20–30%, compared to the level of H0. Moreover, the additional presence of HAp further reduced cytotoxic effect of the analysed extracts. In consequence, the level of the LDH was about 25%, 33%, and 24% higher in H4, H5, and H6 samples, respectively, compared to the control condition. This result indicated that samples H4, H5, and H6 exhibited no cytotoxic effect on hUC-MSCs. 

#### 2.9.3. The Influence of Liquid Extracts of Chitosan on hUC-MSCs Proliferation

**Figure 15:**
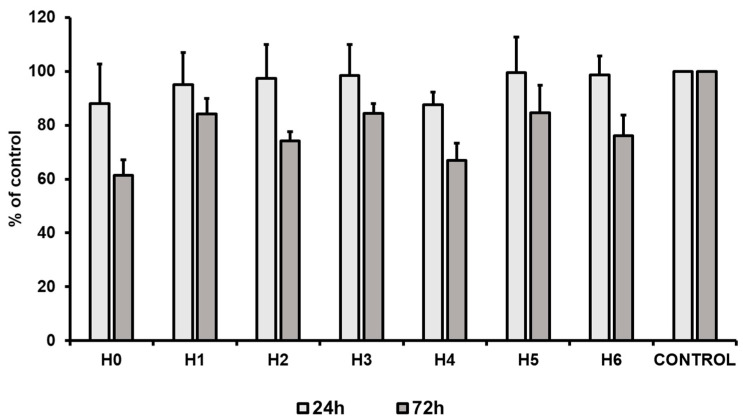
Proliferation of hUC-MSCs (human umbilical cord Wharton’s jelly mesenchymal stem cells) after 24 and 72 h of the cell culture in liquid extracts of chitosan-based samples (H0: chitosan (CS); H1: CS/graphene oxide (GO); H2: CS/poly(ethylene glycol) grafted graphene oxide (GO-PEG); H3: CS/reduced graphene oxide (rGO); H4: CS/GO/hydroxyapatite (HAp); H5: CS/GO-PEG/HAp; H6: CS/rGO/HAp). Data are presented as mean ± SD.

To evaluate the proliferation capacity of hUC-MSCs in a particular liquid extract of chitosan, a proliferation assay was performed. The results revealed that after 24 h of the cell culture a slight decrease of the cell proliferation was observed for chitosan extracts H0 and H4 (about 12% less than the control). Simultaneously, all other tested conditions exhibited no effect on the cell proliferation, that was comparable to the control condition (standard culture medium). However, after 72 h of hUC-MSCs culture a decrease (as compared to the control) in the cell proliferation level was observed in all the tested conditions. The highest decrease was noted for chitosan extracts H0 (CS-only), i.e., about 40% (Figure 15). However, our results revealed that the most suitable for hUC-MSCs propagation was the extract from CS modified with GO-PEG and HAp (H5). We observed that proliferation level for H5 after 24 h, and 72 h was about 100% and 85% of the control condition, respectively. The obtained results are consistent with data presented by Pistone et al. The authors revealed, that modification of CS with HAp may improve proliferation of primary mouse osteoblast cells [35]. Moreover, scaffolds composed of CS, GO and HAp improved MC3T3-E1 cell viability compared to the material consisting only CS and GO. However, this effect is strictly related to the component ratio in the scaffold [36]. Thus, our results indicate that PEG may also improve the cell proliferation and may potentially be used as an additive for preparation of the gradient hydrogels dedicated for tissue engineering. 

## 3. Materials and Methods 

### 3.1. Materials

High molecular weight (M = 600,000–800,000 g/mol) chitosan (CS) with deacetylation degree > 90% and sodium tripolyphosphate (TPP) were purchased from Acros-Organics, Morris Plains, NJ, USA. Lactic acid (LAc, 88%), tannic acid (TAc), NaOH, NaCl, H_2_SO_4_, and reagents needed for the preparation of simulated body fluid (SBF) and phosphate buffered saline (PBS) solutions were obtained from Avantor Performance Materials Poland S.A. H_3_PO_4_, KMnO_4_, NaH_2_PO_2_ and H_2_O_2_ were from Chempur. Polyethylene glycol (PEG 600) and hydroxyapatite (HAp) were purchased from Merck (Darmstadt, Germany) and Chema-Elektromet (Rzeszow, Poland), respectively. All chemicals were of analytical grade and were used without further purification. 

### 3.2. Synthesis of Graphene Oxide (GO), Reduced Graphene Oxide (rGO) and Poly(Ethylene Glycol) Grafted Graphene Oxide (GO-PEG)

Graphene oxide (GO) was prepared by a modified Hummers method [37]. In brief: 3 g of graphite flakes (with the average size of 300–425 µm) were added gradually to a reactor containing 360 mL of concentrated sulphuric acid (H_2_SO_4_; POCH S.A., Gliwice, Poland; 96–98%, pure for analysis p.a.) and 40 mL of orthophosphoric acid (H_3_PO_4_; Chempur, Piekary Slaskie, Poland; p.a.). After that, 18 g of potassium permanganate (KMnO_4_; Chempur, Piekary Slaskie, Poland; p.a.) was slowly added in excess to the graphite weight. Oxidation process was conducted for few hours and it was stopped by the addition of deionized water, and finally 3 mL of perhydrol (30% H_2_O_2_; Chempur, Piekary Slaskie, Poland; p.a.). The water suspension of such obtained graphite oxide was left to sediment. Purifying process was carried out with a microfiltration system. Due to specific shearing forces acting on graphite oxide flakes during the purification, an exfoliation leading to graphene oxide formation occurred. GO in the form of a paste (concentration of 19.2 mg/mL) was prepared by centrifugation.

Reduced graphene oxide (rGO) was prepared through chemical reduction of previously synthesized GO (3 mg/mL; acidified to pH = 0) with the use of a sodium hypophosphite (NaH_2_PO_2_; Chempur, Piekary Slaskie, Poland; p.a.) as a reducing agent. The process was carried out for 4 h in a temperature of 90 °C with a vigorous stirring. The molar ratio of GO:NaH_2_PO_2_ was of 1:2.5 (M_GO_ = 40 g/mol). As obtained rGO was purified with ultrapure water by vacuum filtration. rGO paste (19.2 mg/mL) was prepared by centrifugation.

To obtain GO-PEG composite, PEG-600 (Merck, Darmstadt, Germany); 15% *w*) was added to the GO water dispersion (5 mg/mL) and mixed to obtain homogeneous material. Again, GO-PEG paste (19.2 mg/mL) was prepared by centrifugation.

### 3.3. Preparation of Chitosan Hydrogels Modified with GO, rGO and GO-PEG

Polymer solution was prepared by dissolving 2.5 g of CS powder in 30 mL of 5% LAc solution. Water dispersions of GO and GO-PEG were prepared by sonicating appropriate amount of the pastes in 20 mL of distilled water for 2-h. rGO paste was sonicated under alkaline conditions (pH ~10; adjusted by 0.1 M NaOH addition) to secure colloidal stability of the dispersion thanks to electrostatic repulsion forces between rGO sheets. Next, dispersions of GO, GO-PEG or rGO were introduced to the CS solution (final GFM content: 0.5% to CS weight). After stirring for 24 h, TAc (cross-linker, 10% to CS weight) was added and the whole system was stirred for another 24 h. In the same way, hydrogels modified additionally with HAp (10% to CS weight) were prepared. 

The obtained hydrogel samples were marked as H0–H6, where H0 was the reference CS-only sample and H1–H6 samples were composites modified with GFM (H1-H3 modified with GO, GO-PEG, rGO, respectively) and both GFM and HAp particles (H4–H6). Their composition was summarized in Table 2.

Three cross-linking systems were tested in search for an appropriate solution to obtain gradient hydrogels with desired shape (Table 3). Initially, sodium tripolyphosphate (TPP) was chosen as it is a commonly used physical cross-linker for chitosan injectable hydrogels or microspheres. Cross-linking was carried out at a low temperature (4 °C) to protect frozen solutions from melting too quickly. However, the use of an aqueous TPP solution resulted in a loss of shape and partial disintegration of the hydrogels. An additional bath in 10% NaOH allowed to obtain layered samples with a cross-linked top layer and a liquid interior (2 h in TPP) or empty shells (4 h in TPP). Chitosan amino groups get protonated in an acidic media. This phenomenon was used to extend the cross-linking time with TPP. Then, sodium chloride was introduced into the TPP solution (5% NaCl and 0.5% TPP) to increase the ionic strength. Electrostatic interactions between NH^3+^ ions from CS and Cl^-^ ions from NaCl allowed to extend the cross-linking time to 24-h and finally obtain hydrogels with desired shape and gradient structure.

For cross-linking, the prepared hydrogel solutions (H0–H6) were injected into plastic form (width: 10 mm) and frozen at −20 °C for 24 h. The frozen samples were immersed in the optimized gelling solution (5% NaCl + 0.5% TPP; 24 h) at 4 °C. Finally, the samples were washed with distilled water to remove excess LAc and stored for 24 h before testing.

### 3.4. Characterization

#### 3.4.1. X-Ray Photoelectron Spectroscopy (XPS)

X-ray photoelectron spectroscopy (XPS) characterization of the graphene family materials (GO, rGO and GO-PEG) was carried out using spectrometer (Vacuum Systems Workshop Ltd., Crowborough, East Sussex, UK) with Mg anode (1253.6 eV Kα radiation; 200 W X-ray excitation source; 3 × 10^−8^ mbar vacuum; 15° electron takeoff angle) in the constant analyzer energy mode (22 eV pass energy). The spectra were fitted with Gaussian–Lorentzian peaks with XPSPEAK 4.1 software (Prof. Raymund W.M. Kwok, The Chinese University of Hong Kong).

#### 3.4.2. X-Ray Diffractometry (XRD)

X-ray diffraction analysis of the crystal structure was performed using X’Pert Pro (Malvern Panalytical, Worcestershire, UK) diffractometer with Cu Kα X-ray sources (λ = 1.5406 Å). D-spacing of graphene-based materials was calculated from Bragg equation. 

#### 3.4.3. Attenuated Total Reflection Infrared Spectroscopy (ATR-FTIR)

The chemical compositions of materials were analyzed using Bruker Tensor 27 spectrometer (Bruker, Poznań, Poland) in ATR mode with a diamond crystal. For each sample, spectra were taken in the range of 600–4000 cm^−1^ (64 scans), at 4 cm^−1^ resolution.

#### 3.4.4. Microstructural Analysis

Images of the hydrogels formed in different gelling systems were taken under a digital microscope (VHX-900F; Keyence, Mechelen, Belgium). The morphology of the samples was studied using scanning electron microscope (SEM; Nova NanoSEM 200, FEI, Eindhoven, The Netherlands) with an accelerating voltage of 18 kV. The chemical composition of the hydrogels surface after SBF incubation was analyzed by energy dispersive spectroscopy (EDS; FEI, Eindhoven, The Netherlands method. 

#### 3.4.5. Rheology

Dynamic rheological measurements were performed using rheometer (MCR 302, Anton Paar, Graz, Austria) with stainless steel parallel plates (diameter: 20 mm) geometry, at controlled, constant temperature 25 °C. The viscosity of the solutions was measured in the frequency range between 1 and 100 Hz. The test was carried out to evaluate an influence of graphene materials additives on rheological properties of the polymeric matrix. 

#### 3.4.6. Determination of Cross-Linking Degree

The degree of CS-based hydrogel cross-linking was estimated by ninhydrin assay [38]. 1.5 mg of freeze-dried sample was added to 1 mL of ninhydrin solution and heated in water bath (80 °C) for 20 min. After cooling down to room temperature solution was diluted with 5 mL of isopropanol/water mixture (1:1 *v/v*). The optical absorbance at 570 nm was measured using UV spectrometer (Cecil Instruments Ltd., CE 2502; Cambridge, UK). Glycine in various concentrations was used to prepare a calibration curve. The amount of free amino groups in a sample was proportional to the optical absorbance and allowed to determinate the degree of cross-linking (DCL) of the hydrogel Equation (1):

(1) $$\mathrm{DCL}=\frac{n_{{\mathrm{NH}}_{2\;\left(\mathrm{CS}\right)}}-{\;n}_{{\mathrm{NH}}_{2\;\left(\mathrm{sample}\right)}}}{n_{{\mathrm{NH}}_{2\;\left(\mathrm{CS}\right)}}}\times 100\%$$

where: $n_{{\mathrm{NH}}_{2\;\left(\mathrm{CS}\right)}}$—concentration of NH_2_ in the pristine chitosan (CS) (in mole NH_2_/mole CS), $n_{{\mathrm{NH}}_{2\;\left(\mathrm{sample}\right)}}$—concentration of NH_2_ in the CS-based analyzed sample (in mole NH_2_/mole sample).

#### 3.4.7. Mechanical Properties 

Mechanical properties of the hydrogels were evaluated using a universal mechanical tester (Zwick 1435, ZwickRoell GmbH & Co. KG, Ulm, Germany). The compression test was performed with a 1 mm/min speed until failure. Average values of Young Modulus (E) and compressive strength were calculated from at least three independent measurements. 

#### 3.4.8. In Vitro Chemical Stability

The in vitro degradation of samples was carried out in phosphate buffered saline (PBS) solution at 37 °C. Pieces of the hydrogel samples were immersed in 30 mL of PBS and stored in an incubator for 6 weeks. Once a week, pH of PBS solution was measured and the medium was replaced. The weight loss was quantified over time Equation (2):

(2) $$\mathrm{Weight}\;\mathrm{loss}\;=\frac{\left(W_{0}-W_{t}\right)}{W_{0}}\times 100\%$$

where: W_0_—initial weight of the hydrogel and W_t_—weight of the hydrogel after degradation time. 

#### 3.4.9. Bioactivity Test

Preliminary bioactivity test was carried out using SBF solution. SBF solution with ion concentration similar to those in blood plasma was prepared according to improved protocol described by Bohner [39]. The samples were soaked in SBF at 37 °C for 2 and 4 weeks. The medium was replaced every week. The ratio of sample surface area (mm^2^) and medium volume (mL) was kept constant (10:1) After incubation, the samples were removed from the containers, rinsed with distilled water and dried at 37 °C for SEM/EDS analysis.

#### 3.4.10. Cytocompatibility

##### Isolation and Culture of hUC-MSCs

Mesenchymal stem cells were isolated from the human umbilical cord Wharton’s jelly (hUC-MSCs) by the explant method. Umbilical cords were obtained from The Polish Stem Cell Bank who is a collaborator in the Strategmed 3 project. Cells were cultured in DMEM/F12 (Sigma-Aldrich, Poznan, Poland) medium supplemented with 10 % fetal bovine serum (FBS; Sigma-Aldrich), 100 IU/mL penicillin and 10 µg/mL streptomycin solution (1× Pen/Strep; Thermo Fisher). After 7 days, cells were washed with PBS and adherent fraction of cells was cultured at 37 °C, 5% CO2, and 95% humidity, until they reach confluence. In all experiments, cells between 4–7 passages were utilized.

##### Preparation of Liquid Extracts of Chitosan

Prior to the cytocompatibility evaluation, the hydrogels were cut into 3 mm thick slices. Next, the slices were incubated in 0.5 M NaOH solution for 4 h. NaOH was removed and samples were washed with deionized water three times, each for 10 min. After drying at 37 °C for 1 h, hydrogels were sterilized by incubation in 70% ethanol at room temperature for 30 min and again the samples were dried at 37 °C for 1 h.

According to guidelines included in the PN:EN ISO 10993 Norm, liquid extracts of chitosan were prepared. The 50 mg of chitosan per 1 mL of culture medium (DMEM/F12 without supplements) was used to obtain each chitosan extract. The extraction was performed for 24 h at 37 °C in sterile, chemically inert container by using aseptic techniques. After this time, particular extracts were collected. To remove separated fragments of chitosan, extracts were centrifuged at 300× *g* for 7 min. 

##### Cytotoxicity Test

hUC-MSCs were seeded at 4 × 10^3^ cell density into a 96-well plate (Eppendorf, Hamburg, Germany) in 150 μL of DMEM/F12 medium supplemented with 10 % FBS and 1× Pen/Strep and incubated at 37 °C up to complete adhesion (about 6 h). Then, the culture medium was changed to 150 μL of each chitosan extract supplemented with 2% FBS and 1× Pen/Strep. After 24 h of incubation, the Cytotoxicity Detection Kit (Roche, Basel, Switzerland) was used to evaluate the cytotoxicity of liquid extracts of chitosan on hUC-MSCs. According to the manufacturer’s instruction, plate was centrifuged at 300× *g* for 5 min. Then, the 100 μL of supernatant was transferred to a new 96-well plate (Eppendorf) and the same volume of the LDH-substrate mixture was added to each well. After 30 min of incubation in the dark at the room temperature, the absorbance at 450 and 620 nm was measured by the Multiskan FC microplate photometer (Thermo Scientific, Waltham, MA, USA). hUC-MSCs cultured in DMEM/F12 medium supplemented with 2% FBS medium and 1× Pen/Strep was used as a control. Three independent experiments were performed. Statistical analysis was performed by one-way ANOVA and the Bonferroni test (post-hoc test) using the GraphPad Prism v.5 software.(GraphPad Software, Inc., CA, USA) *p* values less than 0.05 (*p* < 0.05) were considered statistically significant and labelled by an asterisk (*).

##### Proliferation Test

hUC-MSCs at 4 × 10^3^ density were seeded into a 96-well plate (Eppendorf, Hamburg, Germany) in 150 μL of DMEM/F12 medium supplemented with 10 % FBS and 1× Pen/Strep and incubated at 37 °C. When cells adhere to the plate (about 6 h), medium was removed and 150 μL of each chitosan extract supplemented with 10% FBS and 1× P/S was added to each well. Next, cells were incubated at 37 °C, 5% CO_2_, and 95% humidity. The morphological analysis of the hUC-MSCs was performed with the Olympus IX81 microscope (Olympus, Tokyo, Japan) and the Olympus Cell Sens Standard software (Olympus, Tokyo, Japan). The influence of liquid chitosan extracts on hUC-MSCs proliferation was evaluated after 24 and 72 h of cell culture by the Cell Counting Kit-8 (CCK-8, Sigma-Aldrich), according to the manufacturer’s protocol. After 2 h of the CCK-8 incubation with the hUC-MSCs, the absorbance at 450 nm was measured by the Multiskan FC microplate photometer (Thermo Scientific, Waltham, MA, USA). Cells cultured in DMEM/F12 medium supplemented with 10% FBS medium and 1x Pen/Strep were used as a control. Four independent experiments were done. Statistical analysis was performed by one-way ANOVA and the Bonferroni test (post-hoc test) using the GraphPad Prism v.5 software. 

## 4. Conclusions

A freezing–gelling–thawing method was developed to prepare chitosan based hydrogels with a gradient structure following the green-chemistry approach. The use of higher ionic strength cross-linking bath allowed to obtain bulk hydrogel samples with a cross-linking gradient. Composite systems modified with graphene oxide, poly(ethylene glycol)-grafted-graphene oxide, reduced graphene oxide and hydroxyapatite were fabricated and compared. It was shown that the type of the nanofiller introduced into the polymer matrix affects the physicochemical properties of the hydrogels significantly. Modification of GO with PEG favors homogenous distribution of the particles in the hydrogel matrix and strong interchain interaction between CS and PEG, which results in mechanical properties improvement. Graphene derivatives and hydroxyapatite particles interact with chitosan chains and may act as additional cross-linkers. Those interactions are due to the presence of functional groups, as in case of GO and GO-PEG, or may result from a specific arrangement of the sheets, as in case of rGO. The study confirmed that addition of hydroxyapatite, provided that the particles did not agglomerate, improves mechanical properties of the hydrogels. Our results indicate also that GO-PEG together with HAp may improve cell proliferation. Based on the obtained results, gradient hydrogel systems consisting of CS/GO-PEG/HAp were chosen for further evaluation for tissue engineering applications. 

## Figures and Tables

**Table 1:** The main characteristic peaks of the hydrogel components (TPP—sodium tripolyphosphate, HAp—hydroxyapatite, TAc—tannic acid, GO—graphene oxide).

Material	Wavenumber (cm^−1^)	Assignment
**Chitosan**	≈3400	N-H and O-H stretching
	≈2800	C-H symmetric and asymmetric stretching
	1719	C=O stretching in amide I (residual *N*-acetyl groups)
	1576	N-H bending in primary amine
	1450	CH_2_ bending
	1414	CH_3_ symmetrical deformations in amide groups
	1371	N-H bending in amide II
	1219	OH bending
	1120	C-O-C bridge-asymmetric stretching
	1080	C-O stretching
	1070	
	1027	
	855	CH bending out of the plane of the ring
**TPP**	1212	P=O stretching
	1136	O-P=O symmetric and asymmetric stretching
	1090	PO_3_ symmetric and asymmetric stretching
	884	P-O-P bridge asymmetric stretching
**HAp**	1015	P-O asymmetric stretching in PO_4_
**TAc**	1702	C=O stretching
	1443	-C-C_aromatic_- stretching
	1177	C-O stretching
	753	C-C distortion in benzene ring
**GO**	≈3400	O-H stretching
	1725	Carboxyl C=O stretching
	1626	C=C skeletal vibrations
	1391	C-OH deformation
	1073	C-O stretching
**Hydrogels**	≈3400	N-H stretching overlapped with O-H stretching
	1632	N-H bending
	1534	N-O-P stretching

**Table 2:** Composition of the systems used to obtain gradient hydrogels (GO—graphene oxide, GO-PEG—poly(ethylene glycol) grafted graphene oxide, rGO—reduced graphene oxide, HAp—hydroxyapatite; H0: chitosan (CS), H1–H6: CS-based composites).

Sample	GO	GO-PEG	rGO	HAp
**H0 (ref. CS only)**	–	–	–	–
**H1**	0.5%	–	–	–
**H2**	–	0.5%	–	–
**H3**	–	–	0.5%	–
**H4**	0.5%	–	–	10%
**H5**	–	0.5%	–	10%
**H6**	–	–	0.5%	10%

**Table 3:** Gelling system compositions: in the first two attempts samples were immersed consecutively in two solutions for different times, in the third gelling solution was just one (TPP—sodium tripolyphosphate).

Attempt	1st Bath	Time	2nd Bath	Time
1	5% TPP	2 h	10% NaOH	10 min
2	5% TPP	4 h	10% NaOH	10 min
3	5% NaCl + 0.5% TPP	24 h	–	–

## Abbreviations

CS	chitosan
GO	graphene oxide
rGO	reduced graphene oxide
GO-PEG	poly(ethylene glycol) grafted graphene oxide
HAp	hydroxyapatite
PBS	phosphate buffered saline
SBF	simulated body fluid
hUC-MSCs	human umbilical cord Wharton’s jelly mesenchymal stem cells
TPP	sodium tripolyphosphate
